# Cutaneous tactile allodynia associated with microvascular dysfunction in muscle

**DOI:** 10.1186/1744-8069-4-49

**Published:** 2008-10-28

**Authors:** Andre Laferrière, Magali Millecamps, Dimitris N Xanthos, Wen Hua Xiao, Chiang Siau, Marissa de Mos, Christelle Sachot, J Vaigunda Ragavendran, Frank JPM Huygen, Gary J Bennett, Terence J Coderre

**Affiliations:** 1 Department of Anesthesia, McGill University, 3655 Promenade Sir William Osler, Montreal, Quebec, H3G 1Y6, Canada; 2 Alan Edwards Centre for Research on Pain, McGill University, 740 Dr. Penfield Ave, Montreal, Quebec, H3A 1A4, Canada; 3 Department of Psychology, McGill University, 1205 Dr. Penfield Ave, Montreal, Quebec, H3A 1B1, Canada; 4 Faculty of Dentistry, McGill University, 3640 University St, Montreal, Quebec, H3A 2B2, Canada; 5 Department of Medical Informatics, Erasmus University Medical Centre, dr. Molewaterplein 50, 3015 GE Rotterdam, the Netherlands; 6 Department of Neuroscience, Douglas Mental Health University Institute, 6875 LaSalle Boulevard, Verdun, Quebec, H4H 1R3, Canada; 7 Department of Anesthesiology, Pain Treatment Centre, Erasmus University Medical Centre, S. Gravendijkwal 230, 3015 CE Rotterdam, the Netherlands; 8 McGill University Hospital Centre Research Institute, 2155 Guy St, Montreal, Quebec, H3H 2R9, Canada

## Abstract

**Background:**

Cutaneous tactile allodynia, or painful hypersensitivity to mechanical stimulation of the skin, is typically associated with neuropathic pain, although also present in chronic pain patients who do not have evidence of nerve injury. We examine whether deep tissue microvascular dysfunction, a feature common in chronic non-neuropathic pain, contributes to allodynia.

**Results:**

Persistent cutaneous allodynia is produced in rats following a hind paw ischemia-reperfusion injury that induces microvascular dysfunction, including arterial vasospasms and capillary slow flow/no-reflow, in muscle. Microvascular dysfunction leads to persistent muscle ischemia, a reduction of intraepidermal nerve fibers, and allodynia correlated with muscle ischemia, but not with skin nerve loss. The affected hind paw muscle shows lipid peroxidation, an upregulation of nuclear factor kappa B, and enhanced pro-inflammatory cytokines, while allodynia is relieved by agents that inhibit these alterations. Allodynia is increased, along with hind paw muscle lactate, when these rats exercise, and is reduced by an acid sensing ion channel antagonist.

**Conclusion:**

Our results demonstrate how microvascular dysfunction and ischemia in muscle can play a critical role in the development of cutaneous allodynia, and encourage the study of how these mechanisms contribute to chronic pain. We anticipate that focus on the pain mechanisms associated with microvascular dysfunction in muscle will provide new effective treatments for chronic pain patients with cutaneous tactile allodynia.

## Background

Cutaneous tactile allodynia (referred to henceforth as allodynia) is often found in patients with neuropathic pain, and is generally assumed to depend on the sensitization of the central nervous system in response to aberrant activity in damaged peripheral nerves [1]. However, allodynia is also caused by other injuries, such as that produced by ultraviolet radiation, and occurs in association with migraine headache [2] and fibromyalgia [3]. Allodynia is also prominent in complex regional pain syndrome (CRPS) [4], which can be initiated by either soft tissue (CRPS type-I) or nerve (CRPS type-II) injuries. Importantly, what both CRPS subtypes share with UV injury, migraine and fibromyalgia, besides allodynia, are significant vascular abnormalities caused by microvascular dysfunction [5-9]. Also since CNS sensitization, which is critical for allodynia, is more pronounced following deep tissue injury than after cutaneous injury [10], it is possible that microvascular dysfunction in muscle may induce significant allodynia. However, few investigators have assessed vascular abnormalities in the etiology of chronic pain, and none have studied whether microvascular dysfunction in muscle contributes to allodynia. To address these questions, we investigated whether an ischemia-reperfusion (IR) injury produces allodynia in rats, and whether the allodynia is associated with microvascular dysfunction in muscle, and key mechanisms that underlie it. We show that microvascular dysfunction leads to persistent muscle ischemia, a reduction of intraepidermal nerve fibers, and allodynia correlated with muscle ischemia, but not with skin nerve loss. The affected hind paw muscle shows lipid peroxidation, an upregulation of nuclear factor kappa B, and enhanced pro-inflammatory cytokines, while allodynia is relieved by agents that inhibit oxidative stress, nuclear factor kappa B and cytokine activity. Allodynia is increased, along with hind paw muscle lactate, when these rats exercise, and is reduced by an acid sensing ion channel antagonist. Allodynia is also significantly correlated with muscle lactate before and after exercise.

## Results and discussion

**Figure 1:**
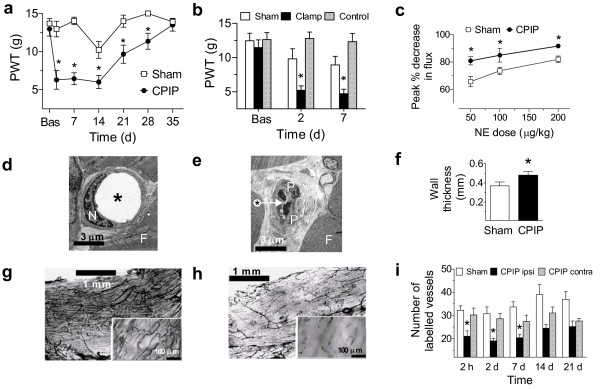
**Allodynia, endothelial cell injury and microvascular dysfunction in muscle induced by hind paw IR injury**. a, 3 h tourniquet IR (CPIP) (n = 12), but not sham (n = 8) treatment, induces a significant reduction in paw-withdrawal threshold (PWT, g) for 4 weeks post-reperfusion (*P < 0.05 compared to baseline (Bas) or sham). b, 3 h clamping of the blood vessels supplying the hind paw (clamp), but not for 5 min (sham), also induces a significant reduction in PWT (g) 2 and 7 days post-reperfusion compared to rats that were only anesthetized (controls) (all groups n = 8) (*P < 0.05, compared to control). c, There are significantly greater dose-dependent norepinephrine (NE)-induced reductions from baseline in blood flow (peak % decrease in flux) in CPIP (n = 8), as compared to sham (n = 13) rats at 2 days post-reperfusion (*P < 0.05) (data from [12]). d, e, Electron micrographs of hind paw digital muscle (HPDM) capillaries from a sham-treated (d) and 7 day CPIP (e) rat (muscle fiber (F), endothelial cell nucleus (N), pericyte (P), lumen (*)). f, Capillary walls are thicker (μm) in CPIP (n = 370 from 4 rats), as compared to sham-treated (n = 206 from 4 rats) rats (*P < 0.05). g, h, Low and higher power (inset) photomicrographs of the India Ink-stained blood vessels in HPDM of the contralateral (g) and ipsilateral (h) CPIP hind paw. i, Number of patent vessels stained with India Ink are significantly reduced in the ipsilateral (ipsi) (n = 6–7), compared to contralateral (contra) CPIP (n = 6–7) and sham (n = 6) HPDM between 2 h and 7 days post-reperfusion (*P < 0.05). All data expressed as mean ± s.e.m.

**Figure 2:**
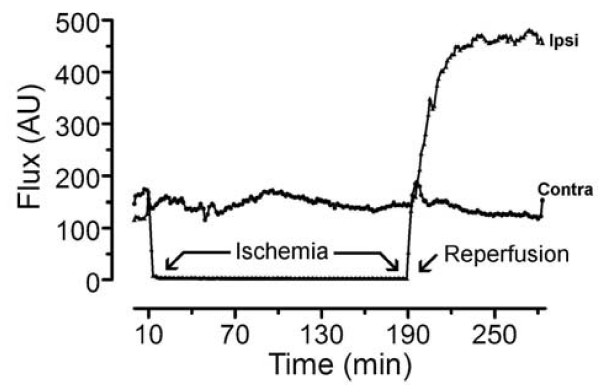
**Bilateral laser Doppler traces during ischemia and reperfusion**. Simultaneous ipsilateral and contralateral representative blood flow measures (arbitrary flux units) in a rat with an O-ring tourniquet placed on the ipsilateral ankle for 3 h between 10 and 190 min. The tourniquet resulted in a complete block of blood flow in the ipsilateral hind paw during the ischemic period, while no change in blood flow was observed in the contralateral hind paw. Reperfusion occurred at 190 min, when the tourniquet was removed, causing an intense and prolonged hyperemia in the ipsilateral hind paw, with only a minor transient change in blood flow in the contralateral hind paw.

**Figure 3:**
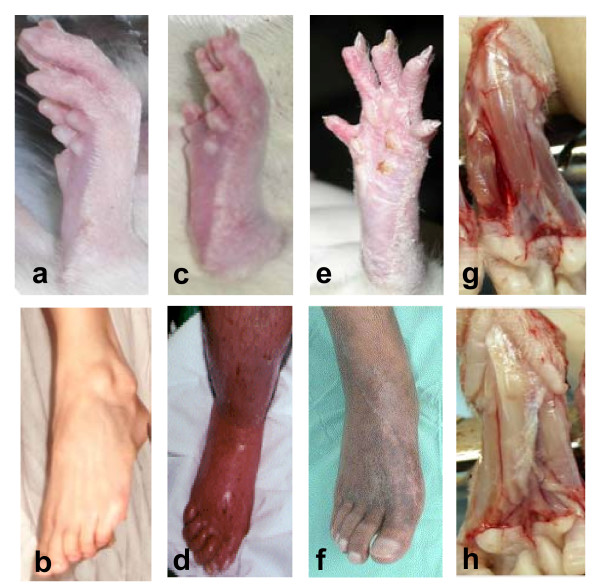
**Photographs of CPIP rat hind paw and CRPS patients' feet**. Comparative photographs of a normal (a), 5 min post-IR hyperemic (c), 24 h post-IR (e), contralateral skinned (g), and 48 hrs post-IR ipsilateral skinned (h) rat hind paw; and normal (b), hot-phase CRPS-I (d), cold-phase CRPS-I (f) foot. Rats are anesthetized in a, c, g and h. Note the similarity between the hyperemic, edematous hot-phase CRPS-I foot and the 5 min post-IR hyperemic hind paw which is also edematous. The 24 h post-IR hind paw appears dry and shinny like the cold-phase CRPS-I foot. The 48 h post-IR ipsilateral skinned hind paw clearly is less well perfused than the contralateral skinned hind paw, comparable to the cyanotic appearance of the cold-phase CRPS-I foot. Human CRPS-I foot photos reprinted with permission from the website of the Reflex Sympathetic Dystrophy Syndrome Association of America. Rat hind paw photos in a, c & e reprinted from Coderre et al., *Pain ***112**, 94–105 (2004) with permission from IASP.

We first tested whether allodynia is exhibited in rats with IR injury of the hind paw. A persistent significant reduction in mechanical paw-withdrawal threshold was observed following a 3 h IR injury induced using a tourniquet at the ankle (P = 0.0001) (Fig. 1a). This procedure produces a complete occlusion of blood flow to the hind paw, followed by prolonged reactive hyperemia (Fig. 2) and edema [11] on reperfusion. In addition to tactile allodynia, rats with what we have called chronic post-ischemia pain (CPIP) exhibit cold allodynia and mechanical hyperalgesia [11], as well as vascular abnormalities [12] that resemble symptoms in CRPS patients (Fig. 3).

To exclude the possibility that CPIP depends on a crush injury of afferent nerves, we examined whether allodynia was also induced after prolonged occlusion of the arteries supplying the hind paw. Mechanical paw-withdrawal thresholds were also persistently significantly reduced in rats whose hind paw blood vessels were occluded for 3 h (P = 0.011) (Fig. 1b), suggesting that allodynia was not caused by tourniquet-induced damage to underlying nerves.

If not nerve crush, it is likely that allodynia depends on IR injury and resultant microvascular dysfunction, which may include arterial vasospasms and capillary slow flow/no-reflow. Arterial vasospasms occur due to a reduction in nitric oxide-induced vasodilatation [13] and hyper-responsiveness of arterial smooth muscle cells to norepinephrine [14]. To determine whether CPIP rats exhibit arterial hyper-responsiveness, we used laser Doppler flowmetry to examine norepinephrine-induced reductions in blood flow in the hind paws of CPIP rats. We found that at 2 days post-perfusion, norepinephrine-induced reductions in CPIP hind paw blood flow were significantly enhanced (P = 0.0062) (Fig. 1c).

Slow flow/no-reflow is a condition where damage to the capillary endothelial cells causes them to swell, and to become clogged with platelets and white blood cells, thus obstructing the passage of red blood cells [15]. We used electron microscopy to assess the morphology of capillaries and endothelial cell thickness in CPIP hind paw muscles. Fig. 1d shows a normal capillary in the hind paw muscle of a sham-operated rat. In contrast, Fig. 1e shows a representative damaged capillary in CPIP hind paw muscle, with swollen endothelial cells and an occluded lumen. As shown in Fig. 1f, the endothelial cells in the hind paw muscle capillaries had significantly thicker (31.5%) cell walls in CPIP rats (P < 0.03971). These findings indicate that CPIP rats exhibit endothelial damage within hind paw muscle capillary beds.

To further characterize slow flow/no-reflow in CPIP rats, we used intra-arterial perfusion of India ink to stain patent blood vessels in the hind paw digital muscle of CPIP rats [16]. As shown in Fig. 1g, most vessels in the muscle capillary bed of the contralateral CPIP hind paw are stained with India ink, indicating that they are patent. In contrast, many vessels in the ipsilateral CPIP hind paw are poorly stained (Fig. 1h). Quantifying this, we found that the ipsilateral CPIP hind paw had significantly fewer patent blood vessels than the contralateral hind paw up to 7 days post-reperfusion (P = 0.0002) (Fig. 1i). This indicates that CPIP rats have persistent capillary slow flow/no-reflow in their hind paw muscles.

**Figure 4:**
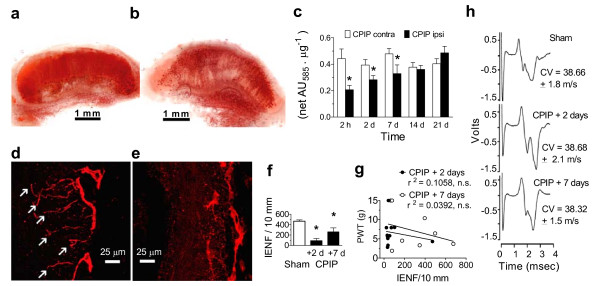
**Muscle and cutaneous nerve alterations induced by hind paw IR injury**. a, b, Photomicrographs of the formazan-stained HPDM of the contralateral (a) and ipsilateral (b) CPIP hind paw, c, Colorimetric assay shows there is significantly less formazan (absorbance (AU) at 585 nm/μg protein) in the ipsilateral (n = 5–7), compared to contralateral (n = 5–7) CPIP HPDM between 2 h and 7 days post-reperfusion (*P < 0.05). d, e, Photo-micrographs of anti-PGP9.5 stained intraepidermal nerve fibers (IENFs, arrows) of sham (d) and 2 day CPIP (e) rat hind paw skin. f, IENF density (number/10 mm) is significantly reduced in day 2 (n = 10) and day 7 (n = 9) CPIP, as compared to sham (n = 11) rats (*P < 0.05). g, Scatter plot of PWT (g) versus the number of IENFs in the ipsilateral CPIP hind paw shows no significant relationship at either 2 or 7 days post-reperfusion. h, Representative voltage traces and analysis summary show there are no significant alterations in sural nerve conduction velocity (CV) between sham (n = 8) and 2 day (n = 6) and 7 day (n = 6) CPIP rats (p > 0.05). All data expressed as mean ± s.e.m.

Muscle is particularly vulnerable to ischemia associated with microvascular dysfunction following IR injury [17]. To assess muscle ischemia, we examined the reduction of triphenyltetrazolium chloride (TTC) to triphenylformazan (formazan red) in CPIP hind paw digital muscle. TTC reduction is indicative of mitochondrial respiration and serves as an indicator of cellular oxygenation [17,18]. Contralateral CPIP hind paw muscle exhibits normal TTC staining, indicated by uniform dark red colour due to effective reduction of TTC to formazan red (Fig. 4a). However, TTC staining is decreased in ipsilateral CPIP hind paw muscle, indicated by the patchy pink-white staining (Fig. 4b). A spectrophotometric assay was used to quantify formazan red in supernatant from homogenized CPIP hind paw muscle tissue. There was significantly less formazan red derived from the ipsilateral, as compared to the contralateral, CPIP hind paw muscle up to 7 days post-reperfusion (P = 0.0314) (Fig. 4c). This indicates that CPIP rats exhibit persistent muscle ischemia associated with reduced mitochondrial respiration.

It is possible that microvascular dysfunction in CPIP rats could induce secondary injury of the distal nerve endings, as previously reported in CRPS-I patients [19]. Thus, we examined intraepidermal nerve fiber (IENF) density in the CPIP hind paw skin using immunocytochemical staining with antibodies to protein gene product 9.5 (PGP9.5), a pan-neuronal marker [20]. Sham rats exhibits normal PGP9.5 skin staining with many IENFs observed in the epidermis (Fig. 4d). However, epidermal PGP9.5 staining is decreased in ipsilateral CPIP hind paw skin, indicating there is a reduction in IENFs (Fig. 4e). Quantifying PGP9.5 staining over the first week after IR injury, we found a significant reduction in IENF density in the epidermis of CPIP hind paw skin (P = 0.0133) (Fig. 4f). However, when we plotted IENF density against mechanical paw-withdrawal thresholds, we found no significant correlation between IENF density and allodynia in CPIP rats at either 2 or 7 days post-reperfusion (P > 0.05) (Fig. 4g). Also, CPIP rats did not exhibit any significant alteration of conduction velocity in the sural nerve (P > 0.05) (Fig. 4h). This is consistent with the absence of a tourniquet-evoked injury to peripheral nerve at the ankle, and similar to findings in CRPS-I patients, who by definition do not have detectable injury at the level of the peripheral nerve.

**Figure 5:**
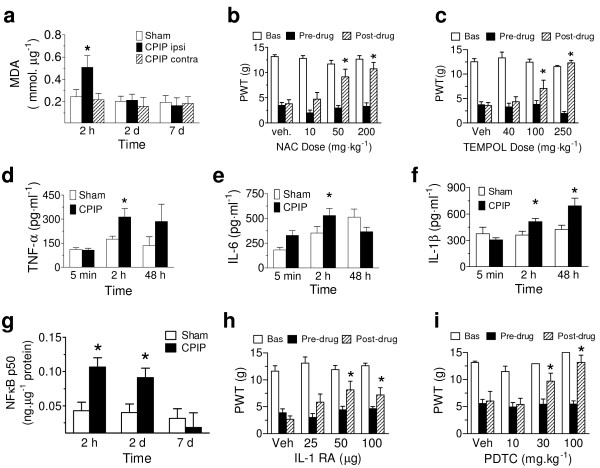
**Lipid peroxidation, cytokines and NFêB induced by IR injury, and effects of inhibitiors on allodynia**. a, Colorimetric assay shows there is significantly greater MDA, mmol/μg) in the ipsilateral (ipsi, n = 7), compared to contralateral (contra, n = 7) CPIP or sham (n = 6) HPDM muscle at 2 hrs post-reperfusion (*P < 0.05). b, c, Reduced pre-drug CPIP PWTs (g) (compared to baseline (Bas)) are significantly and dose-dependently elevated by systemic treatment with NAC (n = 7,6,6,6 for vehicle (Veh), 10,50,200 mg/kg) (b) or TEMPOL (n = 7,6,6,6 for vehicle, 40,100,250 mg/kg) (c) in day 2 CPIP rats (*P < 0.05 compared to pre-drug). d, e, f, ELISA shows there is significantly greater TNFα (pg/ml, n = 9,9,8 for 5 min, 2 h, 48 h) (*P < 0.05)(d), IL-6 (pg/ml, n = 9,9,9 for 5 min, 2 h, 48 h) (*P < 0.05) (e), and IL-1β (pg/ml, n = 9,9,9 for 5 min, 2 h, 48 h) (*P < 0.05) (f) in the HPDM of CPIP, as opposed to sham-treated rats (n = 9,9,8 (d), n = 9,9,9 (e), n = 9,9,9 (f) for 5 min, 2 h, 48 h) at various times post-reperfusion. g, ELISA shows there is significantly greater NFκB p50 (ng/ml protein) in CPIP compared to sham HPDM at 2 hrs (n = 18,15 for CPIP, sham) and 2 days post-reperfusion (n = 18,15 for CPIP, sham) (*P < 0.05), but not 7 days (n = 6,14 for CPIP, sham). h,i, Reduced pre-drug CPIP PWTs (g) (compared to baseline (Bas)) are significantly elevated by intraplantar treatment with IL-1RA (n = 9,4,9,9 for vehicle, 25,50,100 μg) (*P < 0.05 compared to pre-drug) (h) or systemic PDTC (n = 7,9,9,7 for vehicle, 10,30,100 mg/kg) (*P < 0.05 compared to pre-drug) (i) on day 2 post-reperfusion. All data expressed as mean ± s.e.m.

Microvascular dysfunction after IR injury is initiated by oxygen free radicals generated when oxidases that accumulate in ischemic tissue reduce molecular oxygen arriving on reperfusion [21]. We assessed free radical-induced lipid peroxidation in hind paw muscle of CPIP rats, and whether CPIP allodynia is attenuated by the antioxidant N-acetyl-L-cysteine (NAC) or the free radical scavenger 4-hydroxy-2,2,6,6-tetramethylpiperydine-1-oxyl (TEMPOL) given 2 days post-reperfusion when allodynia peaks. Malondialdehyde (MDA), a product of lipid peroxidation, was significantly elevated in ipsilateral, compared to contralateral CPIP hind paws (P = 0.0296) (Fig. 5a), and CPIP allodynia was significantly attenuated by NAC (Fig. 5b) (P = 0.0002) and TEMPOL (P < 0.00001) (Fig. 5c), suggesting a key role of free radicals.

After IR injury, free radicals stimulate the production of pro-inflammatory cytokines [22], following upregulation of nuclear factor kappa B (NFκB) [23]. We used ELISAs to determine the levels of pro-inflammatory cytokines and NFκB in CPIP hind paw muscle, as well as examining the effects of interleukin-1 receptor antagonist (IL-1RA), and an inhibitor of NFκB (pyrrolidine ditiocarbamate, PDTC), on CPIP allodynia 2 days post-reperfusion. The cytokines tumor necrosis factor-α (P = 0.0085) (Fig. 5d), IL-6 (P = 0.0014) (Fig. 5e) and IL-1β (P = 0.0021) (Fig. 5f), and the transcription factor NFκB (P = 0.0073) (Fig. 5g), were all significantly elevated in the CPIP hind paws early after reperfusion, and both IL-1RA (P = 0.0302) (Fig. 5h) and PDTC (P = 0.0009) (Fig. 5i) significantly elevated paw-withdrawal thresholds of CPIP rats, suggesting that NFκB and pro-inflammatory cytokines play a role in CPIP allodynia.

**Figure 6:**
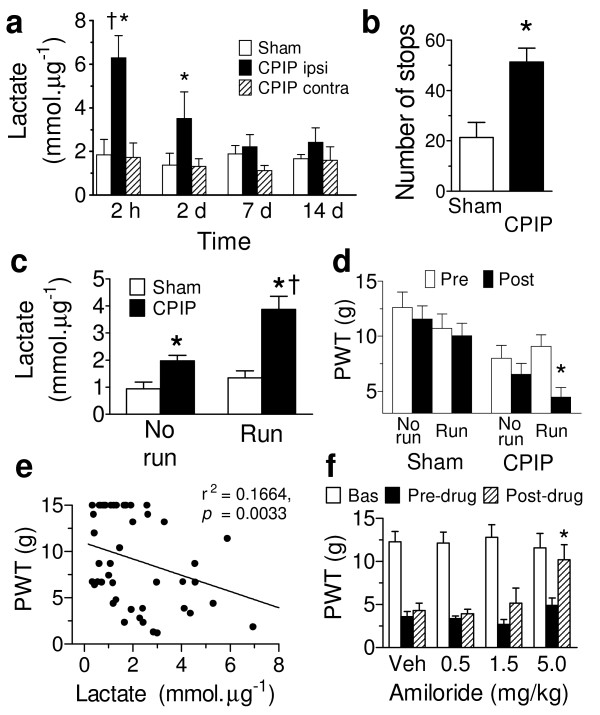
**Relationship between allodynia, muscle ischemia and exercise**. a, compared to sham treatment HPDM lactate (mmol/μg) is increased at 2 hrs (n = 6,6,6 for sham, ipsilateral (ipsi), contralateral (contra)) and 2 days (n = 7,7,5 for sham, ipsi, contra) after hind paw IR injury, and returns to normal at 7 days (n = 7,7,5 for sham, ipsi, contra) and 14 days (n = 6,6,5 for sham, ipsi, contra) post-reperfusion (*P < 0.05). b, 2 days after hind paw IR injury, rats refusal to run results in increased running stoppages in CPIP (n = 7) compared to sham (n = 6) animals (*P < 0.05). c, 7 days after IR injury, HPDM lactate (mmol/μg) levels are significantly increased in unexercised (no run) and exercised (run) CPIP (n = 10), compared to unexercised and exercised sham rats (n = 7), respectively (*P < 0.05). While 20 min of treadmill running does not increase HPDM lactate in sham/run (n = 6) rats (p > 0.05, compared to sham/no run), exercise leads to a further increase in lactate for CPIP/run rats (†P < 0.05 compared to CPIP/no run). d, Although PWTs (g) were lower for all CPIP rats (n = 12,15 for unexercised (no run) and exercised (run)) compared to sham rats (n = 9,9 for unexercised and exercised), there is a significantly greater reduction in PWTs (g) in exercised CPIP rats compared to unexercised CPIP rats (*P < 0.05, Pre vs Post for CPIP run). No significant decrease was observed for unexercised CPIP rats tested twice (p > 0.05, Pre vs Post for CPIP no run). e, scatterplot of PWT (g) vs HPDM lactate (mmol/μg) levels in both exercised and non-exercised sham and 7 day CPIP rats shows a significant inverse linear correlation, indicating that allodynia is directly related to HPDM lactate. f, Reduced pre-drug CPIP PWTs (g) (compared to baseline (Bas)) are significantly elevated by systemic treatment with amiloride on day 2 post-reperfusion (*P < 0.05 compared to pre-drug) (n = 7 for all groups). All data expressed as mean ± s.e.m.

Both ischemia [24] and exercise [25] produce lactate acidosis and muscle pain. We measured lactate levels in the CPIP hind paw muscle for 2 weeks post-reperfusion, and compared lactate levels with paw-withdrawal thresholds. We also assessed whether muscle lactate levels increased, and paw-withdrawal thresholds decreased, in CPIP rats that exercised (running on a circular treadmill). Basal lactate levels were significantly elevated in CPIP rats, particularly early after reperfusion (P = 0.00036) (Fig. 6a). Early post-reperfusion, when lactate levels peaked, CPIP rats showed a strong reluctance to exercise, indicated by significantly increased running stoppages (P = 0.0083) (Fig. 6b). Later post-reperfusion, when basal lactate levels were lower, running resulted in significantly higher lactate levels in the hind paw muscle (P = 0.0215) (Fig. 6c), and significantly lower paw-withdrawal thresholds (P = 0.00135) (Fig. 6d). There was also a significant inverse correlation between lactate level and paw-withdrawal threshold in unexercised and exercised CPIP rats (Fig. 6e). Finally, allodynia in CPIP rats is significantly reduced in rats treated with low doses of the acid sensing ion channel (ASIC) antagonist amiloride (P = 0.02472) (Fig. 6f). These results suggest that muscle ischemia results in the generation of lactate in CPIP rats contributing to pain and allodynia at rest, as well as to exercise-induced pain and increases in allodynia. Importantly, while allodynia was not correlated with cutaneous nerve abnormalities, it was correlated with a measure of muscle ischemia (i.e, lactate), and attenuated by blocking the actions of lactate at ASICs. Parallel to these observations in rats, human CRPS is also marked by increased pain and allodynia after exercise [26]. Although the anti-allodynic effects of amiloride argue for a role of ASICs in CPIP, it is important to note that the effects of amiloride are non-selective as it also affects both epithelial sodium channels and Na+/H+ exchangers [27]. Thus, additional experiments with more selective ASIC antagonists are warranted.

We describe here that allodynia after hind paw IR injury coincides with arterial hypersensitivity to norepinephrine, capillary slow flow/no-reflow and ischemia, and follows increased lipid peroxidation, NFκB, and pro-inflammatory cytokines in muscle tissue. Allodynia is also alleviated by agents that reduce these consequences of microvascular dysfunction, and is directly related to muscle tissue lactate. All these findings indicate that microvascular dysfunction and ischemia in muscle underlies persistent allodynia after IR injury. Our results are consistent with the demonstration that activity in muscle nociceptors is more potent than activity in cutaneous nociceptors in evoking central sensitization [10]. Also, while injecting capsaicin or carrageenan into skin produces allodynia that lasts at most hours, the same injections into muscle induce prolonged central sensitization and cutaneous allodynia lasting up to several weeks [28,29]. Here, IR injury induced alterations in skin nerves, however, unlike the persistent muscle ischemia, these changes were not correlated with allodynia. The finding that allodynia is correlated with muscle lactate (but not skin IENF loss) argues for a more critical role of muscle changes in allodynia. However, we can not dismiss the possibility that the skin IENF loss has some role in the production of allodynia.

**Figure 7:**
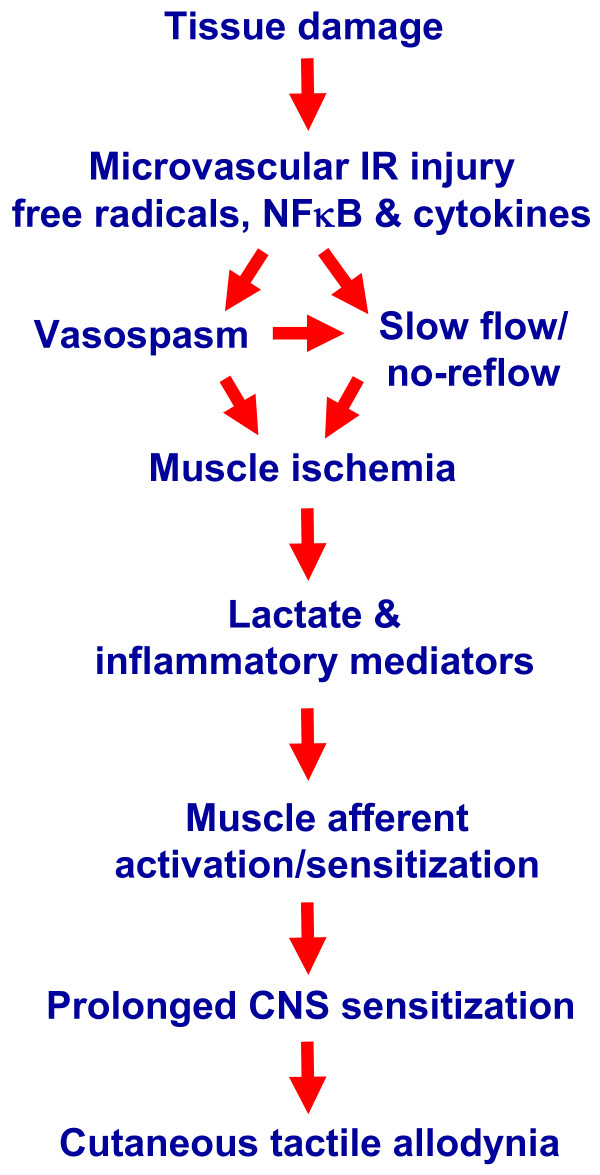
**Schematic diagram indicating proposed mechanisms for initiation/maintenance of cutaneous tactile allodynia after IR injury**. IR injury is generated by oxygen free radicals, NFκB and pro-inflammatory cytokines (TNFα, IL-6, IL-1β) that produce injury to vascular endothelial cells, triggering microvascular dysfunction, including arterial vasospasms and capillary slow flow/no reflow in muscle. Resulting muscle ischemia leads to the generation of lactate and inflammatory mediators which activate ASIC and other receptors on muscle primary afferent fibers. Increased muscle lactate during exercise enhances muscle afferent activation and sensitizes these afferents. Activity in muscle afferents produces prolonged CNS sensitization that results in cutaneous tactile allodynia.

The relationship between microvascular dysfunction in muscle and pathological pain is strengthened by our evidence that allodynia is attenuated by reducing oxygen free radicals, NFκB and pro-inflammatory cytokines, all mediators of IR injury. This is also the first evidence that increased lactate associated with muscle ischemia induces allodynia, at rest or after exercise, in the rat hind paw, and that allodynia after IR injury is reduced with an ASIC antagonist. Importantly, ASIC channels on afferent nerves are tuned to respond to increased lactate [30], and ASIC-3 knock-out mice do not develop the prolonged allodynia or CNS sensitization seen in wild-type mice after muscle injections of acidic saline [31]. Microvascular dysfunction and ischemia in muscle may be critical, but largely ignored, mechanisms of chronic pathological pain, playing a significant role in the etiology of allodynia. See Fig. 7 for a summary schematic of the interpretations of our findings.

## Conclusion

Muscle ischemia and painful symptoms after ischemia-reperfusion injury highlight the importance of microvascular dysfunction in muscle to cutaneous tactile allodynia, or painful hypersensitivity to touch, in chronic pain. Arterial vasospasms, endothelial cell injury and capillary slow flow/no-reflow after ischemia-reperfusion injury induce persistent allodynia dependent on oxygen free-radicals, nuclear factor kappa B, pro-inflammatory cytokines and lactate in muscle. Microvascular dysfunction leads to abnormalities in cutaneous nerves, as well as persistent ischemia in muscle, and that allodynia is significantly correlated with the muscle ischemia, but not with skin nerve changes. Besides nerve cells, pathology in muscle microvasculature may be an important new target for development of therapies for chronic pain.

## Methods

All methods were approved by the McGill Animal Care and Ethics committees.

### IR injury

IR injury was induced in anesthetized rats by placing an O-ring around the ankle (shams only anesthetized) for 3 h as described [11], or by clamping the all blood vessels supplying the hind paw for 3 h (sham rats-5 min) with vascular micro-forceps, followed by reperfusion. Thus, the saphenous artery and the superficial sural artery at the distal margin of gastrocnemius were clamped for 3 h (sham rats-5 min) with microvascular clamps, followed by reperfusion. The adjacent veins were not separated in order to minimize irritation-evoked spasm in the arteries, or stimulation or damage of the sural and saphenous nerves.

### Behavioural studies

Animals were habituated to the testing apparatus 1 day prior to testing and re-acclimatized approximately 30 min prior to any testing. Paw-withdrawal threshold (PWTs) measures were performed as described previously [11]. For drug trials, PWTs were examined at baseline, pre-drug, 20–30 min after administration of NAC, IL-1RA, PDTC, amiloride (Sigma, Oakville, ON), TEMPOL (Tocris, Ellisville, MO), or their saline vehicles, 2 days post-reperfusion. Rats with pre-drug von Frey thresholds below 6 g were randomly assigned into treatment groups. NAC (10, 50, and 200 mg/kg), TEMPOL (25, 100, and 250 mg/kg), PDTC (10, 30, and 100 mg/kg) and amiloride (0.5, 1.5, 5 mg/kg) were injected intraperitoneally, and IL-RA (25, 50 and 100 μg) intraplantarly. The highest doses used for systemic treatments did not result in any significant abnormalities in the rotorod test. All treatment and testing procedures were performed in a blinded manner.

For exercise, rats were placed on a 32.5 cm diameter circular platform bounded on the outside by a 25 cm high Plexiglas cylinder. A second, concentric Plexiglas cylinder delimited the interior side of a 7.5 cm-wide circular alley. The platform was coupled to a variable speed motor and was rotated at 16 rpm (16.3 m/min). Rats were placed on the platform for 20 min, and most ran 326 m in 20 min. A stationary partition hung down above the circular alley producing a barrier when animals ceased to run. A sustained contact with the barrier was counted as a stop. A rapid push on the partition against the pausing rat usually succeeded in re-establishing a sustained running pattern. At 2 days post-reperfusion, CPIP rats exhibited many running stoppages (which were recorded as a measure of exercise-induced pain); post-exercise PWTs were only recorded in 7 day CPIP rats that did not exhibit excessive running stoppages. To assess the effects of exercise on allodynia, we measured baseline PWTs in CPIP and sham-treated rats, then after 30 min rest, rats were exercised for 20 min. Immediately after exercise, each rat was returned to the von Frey test chamber for 15 min habituation, after which PWTs were again recorded. For assays of lactate, the rats were killed by pentobarbital overdose at the end of the second, post-exercise mechanical allodynia test (20 min post-exercise).

### Electron microscopy (EM) & PGP9.5 immunocytochemistry (ICC)

Muscle (EM) or skin (ICC) tissue collected from day 2–7 CPIP or sham-treated rats was processed for EM and for PGP9.5 ICC as described [32].

### Conduction velocity & Laser Doppler flowmetry

Nerve conduction velocity and laser Doppler flux measurements studies were performed in sham-treated and day 2–7 CPIP rats as described [12,33].

### No-reflow

No-reflow was assessed in muscle tissue of day 2–21 CPIP or sham-treated rats by determining the number of ink-filled vessels after hind paw perfusion with India ink as described [16]. Thus, animals were deeply anesthetized with sodium pentobarbital, the ascending vena cava was cut, and 50 ml of 0.1 M, 37°C PBS at pH 7.4, with 1000 U heparin sodium/ml was infused through a cannula placed in the descending aorta, followed by 10.0 ml of 25% (vol/vol) India ink (Pelikan No.17) and 6% gelatin in 0.1 M PBS. The flow rate, catheter gauge and syringe volume were set to produce a mean arterial pressure of 100–110 mm Hg. Plantar muscle samples were collected, fixed in 10% formalin in PBS for 48 h and cryoprotected in 25% sucrose in PBS. 100 μm-thick frozen sections were slide-mounted and digitally photographed under a dissecting microscope. The number of ink-filled vessels which crossed a length-calibrated straight line overlaid on the muscle image at right angle to the orientation of muscle fibers was counted at 2–3 locations on each muscle sample.

### Colorimetric Assays

Mitochrondrial respiration in muscle from 2–21 day CPIP and sham-treated rats was estimated from the reduction of triphenyltetrazolium chloride in muscle slices and homogenized samples as previously described [17,18]. Muscle samples were quickly frozen upon collection and kept at -80 C until processing. Thawed 300 μm-thick sections were incubated in 2% triphenyltetrazolium chloride (TTC; Sigma, St Louis, MO) in 0.05 M PBS for 20 min at 37°C. The sections were then cleared, slide-mounted and examined microscopically. Other samples were homogenized in 0.05 M PBS/0.25 M sucrose. Homogenates were incubated in 0.2% TTC in PBS for 60 min at 37°C in the dark. The formazan was then extracted by incubation in acetone (60 min, 37°C) and centrifugation (10 min at 1000 g). Formazan was estimated by sample absorbance (485 nm) of 200 μl of supernatant, divided by the amount of sample protein (determined by Bradford method). Malondialdehyde and lactate were assayed in muscle samples using colorimetric kits from OxisResearch F(oster City, CA) and BioAssay Systems (Hayward, CA), respectively.

### ELISAs

Animals were sacrificed by decapitation. For the NFκB ELISA, muscle samples were thawed at 4°C and homogenized mechanically in 12.0 μl/mg tissue of RIPA buffer containing 50 mM Tris-HCl, 150 mM NaCl, 1 mM EDTA, 1% Igepal (Sigma, St. Louis, MO), 1% Sodium deoxycholate and 0.1% SDS (Ph 7.4), to which was added a 1% protease inhibitor cocktail (Sigma, St. Louis, MO). Homogenates were centrifuged at 3,000 g for 10 min and the supernatant was collected and processed for nuclear fraction extraction following the recommended procedure of a commercially produced extraction kit (Chemicon Nuclear Extraction Kit, Millipore Corp., Billerica, MA). Nuclear fractions were concentrated by centrifugal filtration using cellulose filters with a 30 kDa cut-off (Microcon YM-30, Millipore Corp., Billerica, MA). The nuclear fraction volume remaining after filtration was collected and diluted in buffer to a final volume of 100 μl. Total sample protein content was determined by the Bradford method (Sigma, St. Louis, MO). NFκB transcription factor measurements were then performed in duplicates using a commercially supplied binding assay (Cayman Chemical, Ann Arbor, MI) and a rabbit polyclonal antibody to the p50 subunit of NFκB (sc-7178, Santa Cruz Biotechnology, Santa Cruz, CA), according to the manufacturer's suggested protocol. NFκB-p50 quantities per well were normalized by dividing the p50 estimate by the total amount of protein measured in the sample.

For the cytokine ELISAs, approximately 200 mg of foot tissue was removed from the plantar surface and dissolved in 750 μl of protease inhibitor cocktail (Sigma, Oakville, ON), 100 μM Amino-n-caproic Acid, 10 μM disodium EDTA, 5 μM benzamidine HCl, and 0.2 μM AEBSF (pH 7.2). Samples were mechanically homogenized, sonicated, and centrifuged at 15,000 RPM at 4°C. The supernatant was used to measure concentrations of the proinflammatory cytokines TNFα, IL-6, and IL-1β using a two-site, rat-specific ELISA as described previously [34]. All samples and standards were assayed in duplicate. Intra- and interassay coefficients of variability were < 7% for all assays and the detection limit for TNFα, IL-1β and IL-6 was ≤ 31.25 pg/ml.

### Statistical Analyses

Statistical significance was determined using one-way or mixed analysis of variance with Fisher's *post hoc *tests, or Pearson correlations.

## Abbreviations

ASIC: acid sensing ion channel; CPIP: chronic post-ischemia pain; CRPS: complex regional pain syndrome; EM: electron microscopic; HPDM: hind paw digital muscle; ICC: immunocyto-chemistry; IENF: intraepidermal nerve fiber; IL: interleukin; IL-1RA: interleukin-1 receptor antagonist; IR: ischemia-reperfusion; MDA: Malondialdehyde ; NAC: N-acetyl-L-cysteine; NE: norepinephrine; NFκB: nuclear factor kappa B; PBS: phosphate-buffered saline; PDTC: pyrrolidine ditiocarbamate; PGP9.5: protein gene product 9.5; PWT: paw withdrawal threshold; TEMPOL: 4-hydroxy-2,2,6,6-tetramethylpiperydine-1-oxyl; TNFα: tumor necrosis factor alpha; TTC: triphenyltetrazolium chloride.

## Competing interests

The authors declare that they have no competing interests.

## Authors' contributions

TJC conceived most of the experiments with input from GJB, AL, DNX and MM. AL carried out the slow flow/no-reflow, TTC, MDA, amiloride behavioural studies and lactate/exercise assays. MM carried out the tourniquet and vessel occlusion behavioral studies and PGP9.5 staining. DNX carried out the laser Doppler studies, cytokine assays, anti-cytokine and anti-oxidant behavioral studies, and participated in the lactate/exercise assays. WHX carried out the nerve conduction velocity studies with help from MM. CSi carried out the electron microscopic studies with help from DNX and MM. MdM carried out the NFκB studies with help from AL and MM. CSa assisted with the cytokine assays. JVR assisted with the amiloride behavioural studies. FJPMH conceived the NFkB experiments along with MdM and TJC. AL, MM, DNX and MdM did the statistical analyses; AL and TJC prepared the figures. TJC and AL co-wrote the paper with assistance from GJB. AL, MM and DNX contributed equally to this work.
